# Primaquine 30 mg/day versus 15 mg/day during 14 days for the prevention of *Plasmodium vivax* relapses in adults in French Guiana: a historical comparison

**DOI:** 10.1186/s12936-018-2378-2

**Published:** 2018-06-19

**Authors:** Audrey Valdes, Loic Epelboin, Emilie Mosnier, Gaelle Walter, Guillaume Vesin, Philippe Abboud, Alessia Melzani, Denis Blanchet, Nicaise Blaise, Mathieu Nacher, Magalie Demar, Felix Djossou

**Affiliations:** 1 Service de Maladies Infectieuses et Tropicales, Centre Hospitalier Andrée Rosemon, Rue de Flamboyants, 97300 Cayenne, French Guiana; 2 Ecosystèmes Amazoniens et Pathologie Tropicale (EPAT), EA 3593, Université de Guyane, Cayenne, French Guiana; 3 Laboratoire Hospitalier Universitaire de Parasitologie et Mycologie, Centre Hospitalier de Cayenne, 97300 Cayenne, French Guiana; 4 Service de Pharmacie, Centre Hospitalier de Cayenne, 97300 Cayenne, French Guiana; 5 Centre d’Investigation Clinique Antilles Guyane, CIC INSERM1424, Centre Hospitalier de Cayenne, 97300 Cayenne, French Guiana

**Keywords:** *Plasmodium vivax*, Malaria, Relapse, Primaquine, French Guiana

## Abstract

**Background:**

The preventive treatment of *Plasmodium vivax* relapse recommended by the World Health Organization is primaquine at a dose of 15 mg/day for 14 days, except for malaria cases from Asia and Oceania. Since 2006, CDC recommends the use of primaquine at 30 mg/day for 14 days. In France, all cases of malaria due to *P. vivax* are treated with 30 mg of primaquine. This systematically increased dosage needs to be evaluated according to epidemiological context. The aim of the study was to compare relapses after 14 days of primaquine at 15 or 30 mg/day.

**Methods:**

All patients treated with primaquine after a vivax malaria episode in French Guiana, between 1 January, 2007 and 1 August, 2016, were studied. Based on the compulsory hospital pharmacy forms for primaquine delivery, adult patients who received 15 or 30 mg of primaquine during 14 days for hypnozoite eradication were included. The recommended dose was initially 15 mg and was changed to 30 mg in 2011. Vivax malaria recurrences within 2 months after primaquine treatment, and vivax malaria recurrences 2–6 months after primaquine in each treatment group were analysed using survival analysis at 2, 3 and 6 months.

**Results:**

Out of 544 patients included, 283 received 15 mg/day and 261 received 30 mg/day of primaquine. At 2 and 3 months after primaquine treatment, the number of recurrences was 7 (2.5%) and 19 (7.3%), and 9 (3.4%) and 15 (5.3%), in the 15 and 30 mg groups (p = 0.51 respectively 0.35), respectively. Within 3 months, the median time to recurrence was 2.05 months in the 15 and 30 mg groups. At 6 months after primaquine treatment, the number of recurrences was 25 (8.8%) and 31 (11.9%) at 15 and 30 mg, respectively (p = 0.24). The median time to recurrence was 2.38 months at 15 mg/day and of 2.64 months at 30 mg/day.

**Conclusions:**

There were no significant differences between primaquine at 15 or 30 mg/day for 14 days in the prevention of *P. vivax* relapses at 2, 3 and 6 months after primaquine treatment in French Guiana.

## Background

The predominant species responsible of malaria in French Guiana *is Plasmodium vivax* [1]. Two other species can be found: *Plasmodium falciparum* and, rarely, *Plasmodium malariae.* Malaria case surveillance is centralized at the regional agency in charge of disease surveillance. However, much of the malaria burden is not detected by the information system because it affects illegal gold miners living deep in the Amazon forest [2–4]. Despite this major reservoir, in the villages of French Guiana, incidence progressively decreased from around 2.5% in the early 2000s to 0.5% in 2011 and even 0.1% in 2016. The proportion of malaria cases caused by *P. vivax* increased from 54 to 70% between 2005 and 2011 [5, 6]. Since 2000, the Roll Back Malaria (World Health Organization, WHO) programme led to a global reduction of malaria cases in South America. However, in the past 2 years, increase of malaria cases has been observed in 9 countries out of 21 endemic countries. On the Guiana shield, in 2016, Guyana, Suriname and French Guiana reported 10,906 cases, 315 cases and 173 cases, respectively [7].

In South America, *P. vivax* remains a hurdle in the eradication of malaria because of its ability to relapse from latent hypnozoites [8]. Differentiating re-infection from relapse is difficult [9, 10]. In French Guiana, relapses are distinguished using the 90-day rule, which states that after a first vivax malaria episode, any vivax episode occurring within 90 days is considered a relapse, whereas those occurring after 90 days are re-infections [11]. Currently, only primaquine is used to treat *P. vivax* relapses worldwide. Safety concerns are important with a risk of lethal haemolysis due to G6PD deficiency [12]. In French Guiana, primaquine requires a special permission from the French authorities in Paris, after ascertaining the absence of G6PD deficiency in a laboratory, often very far from the field. The process can be cumbersome and this may lead to many patients not benefitting from primaquine [13]. WHO recommend treatment for relapse is primaquine, given at 15 mg (or 30 mg in Asia and Oceania)/day during 14 days [14]. Since 2006, center for disease control (CDC) recommends the use of primaquine at 30 mg/day due to an increase of relapses in Southeast Asia [15]. France and UK recommendations also imply the use of primaquine at 30 mg/day during 14 days for all cases of malaria due to *P. vivax*, since 2008 (France) and 2007 (UK) [16, 17]. For malaria cases coming from South America, an increased dose did not seem justified. The increased dose proposed by the CDC for South America is mostly based on case reports of primaquine failure at 15 mg/day (Brazil 1994: 1 case [18]; Colombia 1989: 11 cases [19]; Guyana 1996: 2 cases [20]; Guatemala: 2 cases [21]) and expert opinion [15]. One prospective study tended to show a high relapse rate at 15 mg/day in Colombia [22]: 21 patients out of 87 presented one or more relapses (24.1%). In a prospective study in Brazil in 2001, a lower relapse rate was found with 7 relapses out of 50 patients (14%) treated with primaquine at 15 mg/day [23].

While some trials establish the superiority of the 14-day primaquine regimen versus 5 or 7 day regimen [24], only a few trials compared different primaquine doses given for 14 days [25, 26]. Only one published study was found which compared the efficacy of different primaquine regimens given during 14 days at different doses after an initial treatment by chloroquine. When comparing primaquine 15 mg/day versus 30 mg/day for 14 days, relapse rates were close (6.6% of 322 cases and 8.1% of 317 cases from India, enrolment period 2001–2004) but with more frequent side effects for 30 mg of primaquine/day (1.6 versus 4.1%), which led to frequent treatment interruption in both regimens (40 versus 54%, respectively) [27].

In French Guiana, although French recommendations changed in 2008, the change in practice took place in 2011, which allowed historical comparisons between the 2 drug regimens: 15 mg/day versus 30 mg/day, both given for 14 days to eliminate latent hypnozoites and their potential for relapses.

## Methods

### Study design and participants

A retrospective monocentric study was conducted in French Guiana, a French overseas territory located in northwest South America. Patient data were obtained from merging 3 databases extracted between 1 January, 2007 and 31 July, 2016. The main data were based on primaquine delivery records from Cayenne hospital pharmacy, the main point of primaquine delivery in French Guiana. All patients with positive thin and thick blood smears for *P. vivax* from the Parasitology Laboratory of Cayenne Hospital were compiled. Medical records were collected from patients consulting the Infectious Diseases Department at Cayenne Hospital or in the remote health centres for treatment of a vivax infection. Patients were excluded if they were under 18 years old, had no initial microscopic diagnosis, if they had mixed infections with *P. falciparum*, had a relapse before initiating primaquine or received a different primaquine regimen (adaptation of dose at 22.5 and 7.5 mg/day; treatment delivered for 7 days).

**Fig. 1:**
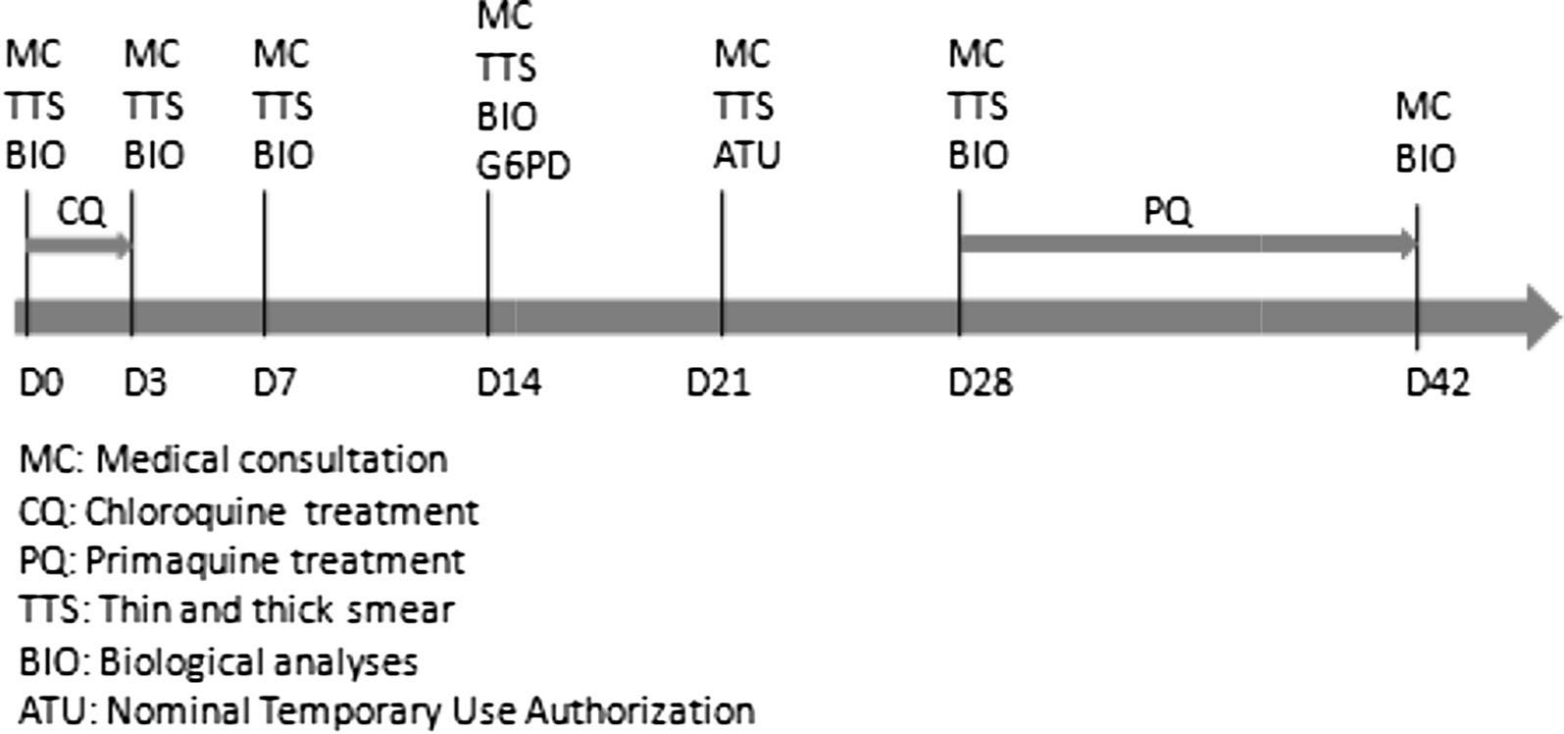
Standard follow-up of patients presenting vivax malaria at Cayenne Hospital

Since 2002, the standard regimen to treat *P. vivax* infection is 3 days of chloroquine (25 mg/kg) followed by 14 days of primaquine, after verifying G6PD test results. Primaquine is only delivered through a nominal temporary use authorization, which requires patients to have tested for G6PD before any treatment delivery. This test is systematically done, usually the second week after acute malaria onset, to avoid underestimation of enzyme activity. The WHO protocol in case of mild G6PD deficiency is not applied in France. The primaquine treatment is usually started on day 28 post-malaria onset. The follow-up is described in Fig. 1. From 2007 to mild 2011, primaquine regimen was used at 15 mg/day during 14 days. Since 2011, primaquine is given at 30 mg/day for 14 days based on the High Commission for Public Health (HCSP) decision to treat imported cases in metropolitan France in 2008 [16].

### Outcome and statistical analysis

Patients following the two regimens were compared at baseline. Only the first infection during the study period was considered.

The occurrence of recurrence of vivax parasitemia in the two groups was described by Kaplan and Meier curves with time starting at the time of primaquine treatment. The time to event analyses included all time at risk from first day receiving primaquine to the earliest event of vivax recurrence or the last date of control data endpoint, 1 February 2017 if the event had not occurred.

A log rank test was performed to compare the two primaquine regimens. Endpoints considered were 2, 3 and 6 months after primaquine course. A delay of 1 month is classically observed between the first malaria access and the use of primaquine, thus the 2 months since primaquine delivery allowed detection of relapses within 3 months since malaria onset. Previous studies in French Guiana have suggested *P. vivax* is related to the Chesson strain, which implies early relapses within 3 months [11]. Events after 2 months since primaquine cure were considered as re-infections. However, the analysis also looked at 3 and 6 months, in the unlikely event that late recurrences would emerge in one of the treatment groups.

The only serious adverse events leading to discontinuation of primaquine in both groups were listed. Statistical description and analyses were done under R 3.4.3.

### Ethic concerns

The study was monocentric based on case records which are authorized by French regulatory authorities. The anonymized database was declared to the CNIL under no 2157449 v 0.

## Results

**Fig. 2:**
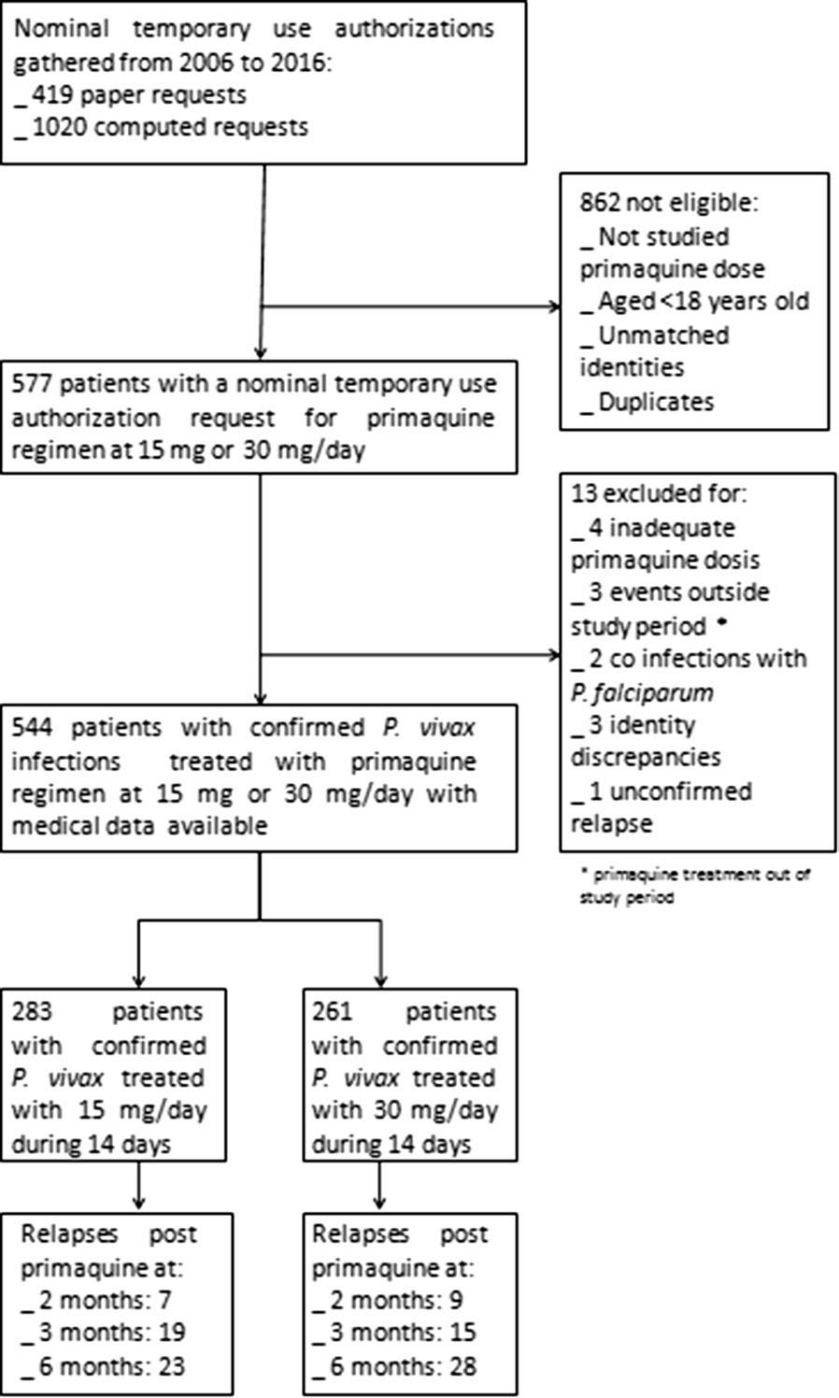
Flow chart of screened patients and outcomes for patients treated with primaquine at 15 or 30 mg/day for 14 days in French Guiana

The main database was composed of 1439 nominal temporary use authorizations for primaquine obtained at the pharmacy. From medical records, the term *P. vivax* was used 1017 times as first reason for medical consultation. From the laboratory the analysis of thick and thin smears positive for either *P. vivax* and/or *P. falciparum* retrieved 3181 specimens. By merging the three databases, 557 patient files were kept. Thirteen additional patients were excluded. Reasons and steps of exclusion are described in the flow chart (Fig. 2).

No major adverse event due to primaquine leading to discontinuation occurred. The baseline characteristics (Table 1) were different in the two groups.

**Table 1:** Main characteristics at baseline of patients treated with primaquine at 15 or 30 mg/day for 14 days in French Guiana

Population characteristics	Primaquine regimen		p-value
	15 mg/day, n = 283	30 mg/day, n = 261	
Age (mean in years ± sd)	36.8 ± 13.5	33.8 ± 10.6	0.004
Sex ratio (male/female)	3.0	5.5	0.02
Weight (mean in kg ± sd)	71.7 ± 12.6	76.9 ± 10.4	< 0.001
Weight (range in kg)	40–119	41–130	

Out of the 544 cases included, 283 patients were treated with 15 mg/day before 2011 and 261 patients were treated with 30 mg/day after 2011 except for 12 cases that received 15 mg/day after 2011. At 2 months after primaquine treatment, the number of recurrences was 7 (2.5%) at 15 mg/day and 9 (3.4%) at 30 mg/day. The log rank test showed no significant difference between the two groups (*p* = 0.51). The median time before a relapse event at 15 mg/day was of 1.52 and 1.58 months at 30 mg/day. The mean weight of patients with a relapse at 2 months was 77.9 ± 11.2 at 15 mg/day and 73.9 ± 11.8 at 30 mg/day (p = 0.49). At 3 months after primaquine treatment, the number of recurrences was 15 (5.3%) at 15 mg/day and 19 (7.3%) at 30 mg/day. The log rank test shows no differences between the two groups (*p* = 0.35). The median time before recurrence at 15 mg/day was 2.05 months, the median time was also 2.05 months at 30 mg/day. The mean weight of patients with a recurrence within 3 months was 73.8 ± at 15 mg/day and 75.7 ± at 30 mg/day (p = 0.61). At 6 months after primaquine treatment, the number of vivax infections was 23 (8.8%) at 15 mg/day and 28 (10.7%) at 30 mg/day. The log rank test showed no significant difference between the two groups (*p* = 0.29). The median time before vivax infection at 15 mg/day was 2.34 and 2.56 months at 30 mg/day.

**Fig. 3:**
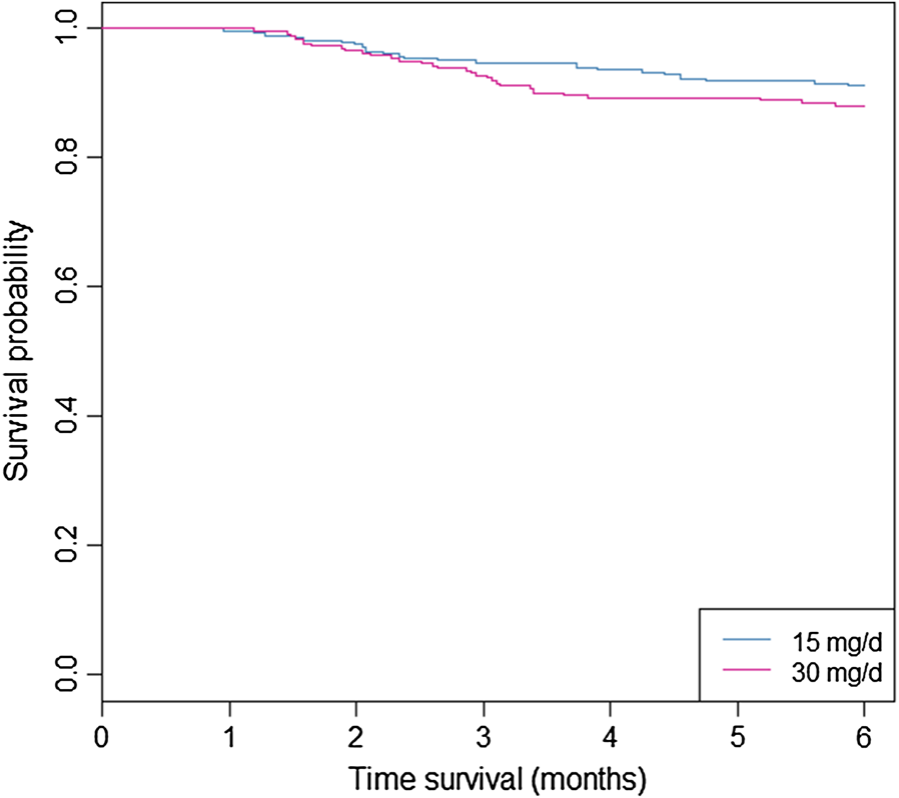
Representation of relapses by Kaplan and Meier curves for patients treated by primaquine 15 or 30 mg/day for 14 days in French Guiana

**Fig. 4:**
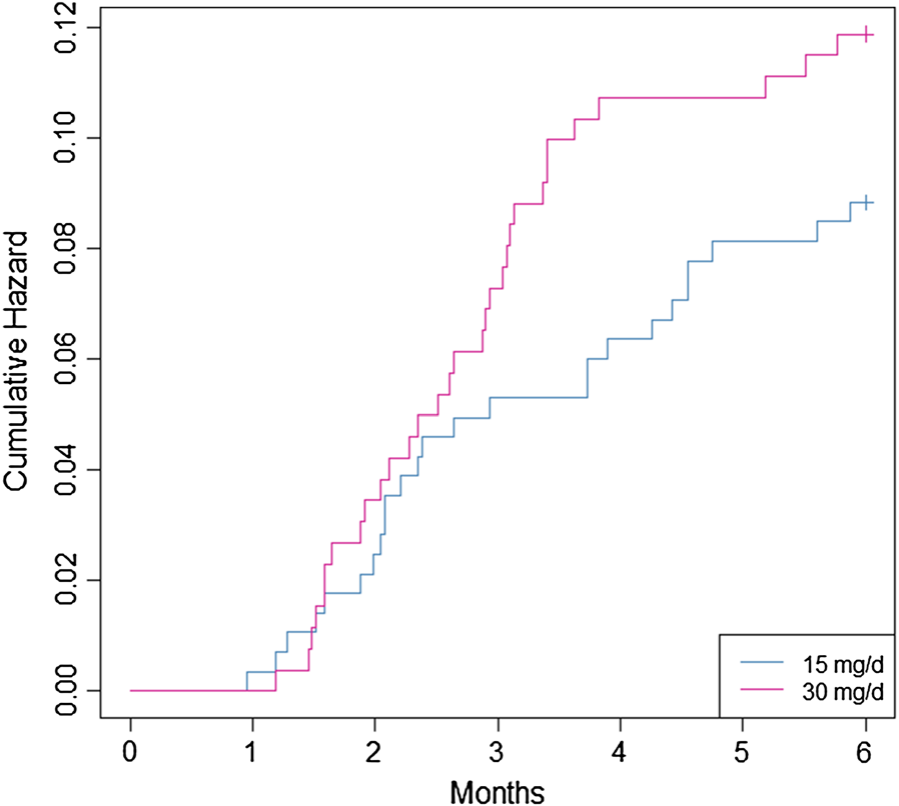
Representation of relapses risks for patients treated by primaquine at 15 or 30 mg/day for 14 days in French Guiana within 2 months post primaquine treatment

The Kaplan and Meier curves are presented in Fig. 3. The cumulative hazard ratio of presenting a relapse at 15 and 30 mg/day are presented in Fig. 4.

## Discussion

The aim of the study was to find out if, in French Guiana, the number of vivax recurrences was higher with primaquine 15 mg/day than with primaquine 30 mg/day. The present results suggest that there was no difference between the two regimens. The present study has several limitations. The first one is that it is a historical comparison. The differences between the two groups at baseline were presumably explained by the evolving epidemiological context in French Guiana with fewer cases each year due to vector control efforts and the distribution of impregnated bed nets in the villages. Although the situation has markedly improved in the village populations, the situation is contrasted with an increase of malaria cases in illegal gold miners and military personnel who are sent into the forest to destroy the gold mining sites.

A second limitation is that treatment was unsupervised, which raises the possibility that the two groups had different adherence levels due to different dosages and frequency of side effects. However, no serious adverse events leading to primaquine discontinuation was observed. The main major adverse event is prevented by the systematic test for G6PD deficiency. In other studies testing for G6PD deficiency, only minor adverse events were reported and they did not lead to primaquine discontinuation [28].

In the present study, patients were not followed after their primaquine regimen, thus treatment adherence was only assessed during the basic follow-up for patients at Cayenne Hospital. For unsupervised primaquine treatment, it has been shown that patients with a regimen for over 5 days had poor adherence [29]. There were no significant patient weight differences between patients with a vivax recurrence, which could have biased the analysis [30]. The absence of difference could have been explained by the fact that the two dosages were equivalent in their capacity to destroy hypnozoites. However, an alternative explanation would be that the greater efficacy of the 30-mg dosage was offset by a greater proportion of patients discontinuing the treatment, thus leading to a lack of difference between groups. However, there was no arguments for differences in adherence, which is in favour of the first hypothesis.

Perhaps it may be more efficient to use primaquine for 14 days, as shown in a systematic review in 2013 [24] where 15 mg/day for 14 days appeared to be better than 5- or 7-day regimens. There were no trials at the time of this review comparing 15 and 30 mg/day for 14 days. There was only one published study [27]. In studies performing sub-analyses to try to answer this question, no differences were observed between 15 and 30 mg/day but without a high proof level.

Other studies made different comparisons: 3 arms with < 2.5; 2.5–5; > 5 mg/kg with an end point at 1 month, which makes comparisons with French Guiana difficult [31].

Historical studies showed that Korean strains of *P. vivax* were exquisitely sensitive to 15 mg primaquine regimen, whereas the Chesson strain from New Guinea was not and required 30 mg daily dosing to achieve good efficacy [32]. If local strains were sensitive to the 15 mg regimen, then one would expect to see little advantage to using 30 mg over 15 mg. This may be specific to *P. vivax* on the northeastern coast of South America.

French Guiana, a French territory, complied with French recommendations that mostly consider imported malaria cases in France. The present study suggests that this may not be warranted in the Amazonian epidemiological context. Furthermore, since mass G6PD testing has not been performed in French Guiana, the primaquine course is delayed by weeks due to mandatory G6PD testing. Earlier primaquine treatment may avoid some cases of relapses taking place in the first month and may avoid losing some patients, often living in remote places, who do not come back a month later for primaquine treatment. This underlines the need of adapting strategies in malaria-endemic countries compared to non-endemic countries.

## Conclusions

Despite its methodological limitations, this study suggests there was no difference between the use of primaquine at 15 mg or at 30 mg/day given for 14 days to prevent relapses of vivax malaria in French Guiana. This non-randomized historical assessment suggests that *P. vivax* strains occurring in French Guiana may be equally sensitive to 15 mg daily primaquine for 14 days relative to the 30 mg regimen. These findings are a first step and an argument for considering formal evaluations of the two dosages.
